# Les surdosages aux antivitamines K à Dakar: aspects épidémiologiques, cliniques et évolutifs

**DOI:** 10.11604/pamj.2016.24.186.8256

**Published:** 2016-07-01

**Authors:** Khadidiatou Dia, Simon Antoine Sarr, Mohamed Cherif Mboup, Djibril Marie Ba, Pape Diadie Fall

**Affiliations:** 1 Service Cardiologie Hôpital Principal de Dakar, Sénégal; 2 Clinique Cardiologique CHU Aristide Le Dantec de Dakar, Sénégal; 3 Service de Cardiologie Hôpital Militaire de Ouakam Dakar, Sénégal

**Keywords:** Antivitamines K, AVK, hémorragies, surdosage, INR, vitamine K, Vitamin K antagonists, VKA haemorrhages, overdose, INR, vitamin K

## Abstract

Les antivitamines K (AVK) sont largement utilisées dans la prévention et le traitement curatif des accidents thromboemboliques. Les objectifs de ce travail étaient de décrire les aspects épidémiologiques, cliniques et évolutifs des surdosages en AVK et d’en déterminer les facteurs hémorragiques. Il s’agit d’une étude descriptive, transversale monocentrique réalisée à l’Hôpital Principal de Dakar. Tous les patients qui présentaient un INR supérieur à 5 étaient inclus. Etaient étudiés le sexe, l’âge du patient, l’AVK utilisé, l’ancienneté de sa prise, les indications, la valeur de l’INR, les médicaments associés, la présence d’hémorragie, la prise en charge immédiate et l’évolution. Nous avons inclus 154 patients. L’acénocoumarol était l’AVK le plus prescrit. Le sexe ratio était en faveur des femmes. L’âge moyen était de 63 ans. Le surdosage était asymptomatique chez 43% des patients. Les hémorragies étaient représentées principalement par des gingivorragies, épistaxis. Des hémorragies majeures étaient présentes chez 8,6% des patients représentées par des mélénas chez 6 patients (3,9%), un hématome musculaire profond chez 2 patients (1,3%) et des hématomes cérébraux intra-parenchymateux chez 2 autres. Deux patients présentaient un collapsus cardiovasculaire avec déglobulisation. Une prise d’AINS était notée chez 21% des patients. Les AVK ont été transitoirement arrêtés chez tous les patients. La mortalité était de 2% par hémorragie intracrânienne. La réduction des surdosages aux AVK passe par une maitrise par le personnel soignant des facteurs de surdosage et par bonne éducation thérapeutique des patients.

## Abstract (en)

Vitamin K antagonists (VKA) are widely used for the prevention and curative treatment of thromboembolic events. This study aims to describe the epidemiological, clinical and evolutionary aspects of overdose in Vitamin K antagonists administration and determine its hemorrhagic factors. We conducted a monocentric cross-sectional descriptive study at the Principal Hospital in Dakar. All patients with an INR greater than 5 were included. We studied patients’ gender and age, VKA used, drug use period, indications, INR value, associated drugs, presence of hemorrhage, immediate management and evolution. We enrolled 154 patients. Acenocoumarol was the most prescribed VKA. Sex ratio favoured women. The average age was 63 years. Overdose was asymptomatic in 43% of patients. Hemorrhagic symptoms were mainly represented by gingival bleeding, epistaxis. Major bleeding episodes were found in 8.6% of patients and they were represented by melena in 6 patients (3.9%), deep muscle hematoma in 2 patients (1.3%) and intracerebral parenchymal hematoma in 2 patients. Two patients had cardiovascular collapse associated with deglobulisation. Nonsteroidal anti-inflammatory drugs (NSAIDs) assumption was noted in 21% of patients. VKA assumption was suspended transiently in all patients. Mortality was 2%, due to intracranial hemorrhage. The reduction in VKA overdose requires caregivers to manage overdose factors and provide proper patient education.

## Introduction

Les antivitamines K (AVK) ont montré leur efficacité dans la prévention et le traitement des accidents thromboemboliques. Ils nécessitent cependant une utilisation minutieuse à bonne dose; en effet, sous-dosés, ils exposent à des complications thrombotiques, mais, en surdosage, ils peuvent être à l’origine d’hémorragies qui demeurent une préoccupation constante. En Afrique et plus particulièrement au Sénégal, peu d’études ont été consacrées aux surdosages aux AVK qui sont pourtant, dans la plupart des pays africains, les seuls anticoagulants oraux disponibles. Il nous a alors paru judicieux de réaliser cette étude dont l’intérêt était de déterminer la prévalence de ces surdosages et les facteurs de risque; nous permettant ainsi, dans notre pratique courante, d’éviter certaines situations pouvant être sources de surdosages. Les objectifs de ce travail étaient de déterminer les aspects épidémiologiques, cliniques et évolutifs des surdosages liés aux AVK à Dakar et dans un second temps d’en déterminer les facteurs de risque.

## Méthodes

Nous avons effectué une étude descriptive et transversale monocentrique au service de cardiologie de l’hôpital Principal de Dakar de Février 2011 à Octobre 2012 puis de Novembre 2014 à Mai 2015. Etaient inclus tous les patients hospitalisés ou suivis en ambulatoire qui étaient sous AVK et qui présentaient un International Normalized Ratio (INR) supérieur ou égal à 5. Pour tous ces patients, des fiches étaient remplies comportant les données suivantes: sexe, âge, indication de l’anticoagulation, nom de l’AVK, l’ancienneté de prise du traitement, les médicaments associés, l’existence d’une insuffisance cardiaque, d’une insuffisance rénale, l’existence de signe hémorragique, d’une instabilité hémodynamique, la valeur de l’INR, du taux d’hémoglobine, la prise en charge thérapeutique immédiate et l’évolution.

## Résultats

Cent cinquante quatre (154) patients ont été ainsi colligés. Ils représentaient 19,6% des patients sous AVK suivis durant cette période. Le sex-ratio était de 61H/93F. L’âge moyen était de 63± 13 ans (extrêmes 24 et 85 ans). Cinquante huit pour cent (58%) des patients avaient plus de 60 ans et 6% avaient plus de 80 ans (Tableau 1). L’acénocoumarol était l’AVK le plus prescrit (92%) avec une dose moyenne de 4,7mg/j. l’INR était en moyenne à 5,9. Dix huit pour cent (18%) des patients avaient un Taux de Prothrombine (TP) incoagulable. Prés du tiers des patients (32%) était sous anticoagulant depuis plus d’un an. Vingt sept pour cent (27%) des patients prenaient des AVK depuis moins de 3 mois. Chez 34% des patients, aucun facteur de surdosage n’a été retrouvé. Une insuffisance cardiaque était présente dans 19% des cas, une insuffisance rénale avec une clairance ≤ 60 mL/mn était présente chez 6% des patients. Une prise concomitante d’AINS en comprimé ou en pommade était retrouvée chez un patient sur cinq (21%). Une association à l’amiodarone était retrouvée dans 15% des cas, d’antibiotique chez 8% des patients, d’antifongique et d’antiagrégants plaquettaires dans 7% des cas. Les principales indications étaient représentées par les troubles du rythme auriculaire (42%), les cardiopathies emboligénes (29%), la maladie veineuse thromboembolique (MVTE) dans 20% des cas et les prothèses valvulaires mécaniques chez 9% des patients (Tableau 1). Le surdosage était asymptomatique chez 42,8% des patients. Le surdosage était hémorragique chez 57,2% des patients soit 11% de l’ensemble des patients sous AVK. Les hémorragies étaient représentées principalement par des gingivorragies dans 18% des cas, des hémoptysies (11%), épistaxis (9%). Les saignements majeurs étaient notés chez 12 patients soit 7,8% des patients en surdosage et étaient représentés par des mélénas chez 6 patients, un hématome musculaire profond chez 3 patients et des hématomes cérébraux intra-parenchymateux chez 3 patients. Deux patients présentaient un collapsus cardiovasculaire avec déglobulisation (Tableau 2). Les AVK ont été arrêtés transitoirement chez tous les patients puis réintroduits à plus faible dose dés diminution de l’INR. Un INR de contrôle était réalisé systématiquement à 24H et 48 H chez tous les patients hospitalisés et à 72 heures chez les autres. Deux patients ont bénéficié d’administration de macromolécules et de transfusion sanguine isogroupe isorhésus. La vitamine K a été administrée chez 69% des patients à la posologie de 2 mg en IVD. Chez 4 patients, elle était donnée à 5 mg. L’évolution était favorable chez 97% des patients. La mortalité était de 2% par hémorragie cérébrale.

**Tableau 1:** Caractéristiques démographiques et médicales des patients

Variables	n	%
*Sex-ratio*	61H /93F	
*Classes d’âge*		
20-39 ans	28	18,2
40-59 ans	36	23,4
60-79 ans	80	51,9
80-99 ans	10	6,5
*AVK utilisé*		
Acénocoumarol	142	92,2
Fluindione	12	7,8
*Indications des AVK*		
Troubles du rythme	65	42,2
Cardiopathies emboligénes	45	29,2
MVTE	31	20,1
Prothèses valvulaires mécaniques	13	8,5
*Ancienneté prise AVK*		
0-3 mois	43	28
3-6 mois	26	16,9
6-9 mois	19	12,3
9-12 mois	17	11
˃ 12 mois	49	31,8
*Insuffisance cardiaque*	30	19,4
*Insuffisance rénale*	9	5,8
*Médicaments associés*		
AINS	32	20,8
Amiodarone	22	14,3
ATB	13	8,4
Antifongiques	11	7,1
AAP	11	7,1
IPP	9	5,9
Total	98	63,6

MVTE: maladie veineuse thrombo-embolique; AINS: anti-inflammatoires non stéroïdiens; ATB: antibiotiques, AAP: antiagrégants plaquettaires, IPP: inhibiteur de la pompe à protons

**Tableau 2:** Présentation clinique des surdosages

Symptômes	n	%
Asymptomatique	66	42,9
Gingivorragies	29	18,8
Hémoptysies	18	11,7
Epistaxis	14	9
Hématuries	7	4,5
Ecchymoses	8	5,2
Mélénas	6	3,9
Hématome musculaire profond	3	2
Hématome cérébral	3	2

## Discussion

Dans notre étude l’incidence des surdosages aux AVK est de 19,6%. Elle se rapproche de celles trouvées dans d’autres études [1]. Une enquête réalisée par l’Afssaps auprès de 436 laboratoires d’analyse médicale montre que lorsque la fourchette d’INR est comprise entre 2 et 3, seulement 43% des INR sont corrects et 33% sont trop élevés; lorsque cette fourchette se situe entre 3 et 4,5 seuls 36% des INR sont dans l’intervalle cible et 16% sont trop élevés [2]. Ceci constitue un véritable problème de santé publique en France où les surdosages hémorragiques par AVK sont la première cause d’hospitalisation pour cause iatrogène en France. Au Royaume uni ils viennent en troisième position [1]. Dans notre série, les surdosages sont fréquemment observés chez les patients âgés. La moyenne d’âge est de 63 ans et 58% de nos patients ont plus de 60 ans. Dans une étude tunisienne le surdosage est retrouvé chez une population plus jeune (55 ± 14 ans) [3]. Mais en général, dans la littérature la moyenne d’âge est plus élevée entre 71 et 80 ans sans doute liés au vieillissement de la population générale occidentale [4]. Plusieurs hypothèses sont émises: une plus grande sensibilité au traitement par AVK et une plus grande variabilité dans la réponse anticoagulante. Cette sensibilité accrue est parfois multi-factorielle: dénutrition, diminution de la masse musculaire et modification du transport et de la distribution de l’AVK, présence fréquente d’une insuffisance rénale, interactions médicamenteuse chez cette population souvent poly-médiquée, mauvaise compliance au traitement ou parfois incompréhension des posologies [5]. Le sex-ratio dans notre étude est en faveur des femmes 93F/61H. Le sexe féminin pourrait être un facteur de risque de surdosage d’après certains auteurs [6, 7]; Pour d’autres c’est plutôt un facteur de risque d’hémorragie majeure [8] et concluent qu’elles nécessitent des doses plus faibles que les hommes. Le mécanisme de cette sensibilité des femmes au traitement par AVK n’est pas clairement élucidé. Certaines hypothèses ont été émises notamment les différences de taille, de graisse hépatique [6, 8]. Mais toutes ces études ont été réalisées avec la warfarine et non avec l’acénocoumarol ou le fluindione. Nous avons trouvé dans notre travail que prés de la moitié des surdosages était asymptomatique (42,8%). Ailleurs les proportions rapportées sont moins importantes entre 21 et 32% [3]. Cette différence est probablement liée à l’INR moyen de notre série relativement bas 5,9 comparé à celui rapporté dans ces séries entre 9,2 et 10 [2, 3]. En ce qui concerne la présentation clinique des surdosages, la littérature est très hétérogène. Les hémorragies les plus fréquemment retrouvées sont les gingivorragies, les épistaxis, comme dans notre étude, dans d’autres séries les hématuries sont les plus fréquentes [3]. Les hémorragies digestives et cérébrales sont plus rarement retrouvées entre 2,4 et 10% souvent associées à une mortalité plus importante [3, 9, 10, 11]. Les hémorragies intra-craniennes survenant sous AVK sont fatales entre 60 et 80% [12]. Dans notre série elle est de 100 % pour les hémorragies cérébrales. Une interaction médicamenteuse est présente chez plus de la moitié de nos patients (63%). Dans la série de Deal, 30% des surdosages étaient liés à la prise concomitante d’autres médicaments [4]. En tête de file, les AINS sont en cause dans notre série chez un patient sur cinq qu’ils soient administrés par voie locale (pommade, gel) ou orale. Cette interaction potentialisatrice a été également retrouvée par d’autres auteurs souvent en automédication [3, 11, 13]. L’amiodarone, les antibiotiques et les antifongiques sont associés à un INR élevé dans une proportion moindre dans notre série mais sont également retrouvés dans certaines études [7]. Les antiagrégants sont également incriminés remettant en cause la pertinence de son association avec les AVK dans certaines situations. Chez certains patients notamment coronariens, une réduction des doses d’AVK et la réalisation d’INR rapprochés est vivement préconisée [9]. La période initiale du traitement parait associée, dans notre étude, à un risque majoré de surdosages. En effet, il est noté chez un tiers de nos patients (28%) dans les 3 premiers mois du traitement. Ceci est rapporté par de nombreux auteurs qui trouvent une fréquence accrue de surdosages au cours des 90 premiers jours d’initiation du traitement souvent liée au fait que l’INR est moins stable en début de traitement mais aussi que la détermination de la posologie peut être problématique chez certains patients [9, 14, 15]. Pour d’autres auteurs, seul le premier mois d’initiation du traitement est concerné [3, 5, 7]. Cependant nous avons également noté dans notre série que 32% des patients prenaient des AVK depuis plus de 12 mois, ceci pourrait être lié au fait que ce sont des patients ayant un état général parfois plus précaire avec plusieurs comorbidités, une pathologie sous-jacente plus évoluée et prenant souvent plusieurs thérapeutiques. Des comorbidités sont présentes chez certains de nos patients avec notamment une insuffisance cardiaque chez 19% liée probablement au fait que cet état s’associe souvent à une hypocoagulabilité spontanée du fait de l’insuffisance hépatocellulaire. Une insuffisance rénale est retrouvée chez 6% de nos patients, son incrimination dans la survenue de surdosage est relatée par certains auteurs qui l’expliquent par le fait que les AVK ayant une élimination rénale, restent stockés dans l’organisme en cas d’insuffisance rénale et être ainsi à l’origine d’INR élevés. La mortalité dans notre étude est de 2%. Dans la littérature, elle varie entre 4,1 et 6,5% principalement par hémorragies digestives et cérébrales [16]. Soixante pour cent (60%) des hémorragies intracrâniennes sous AVK sont fatales [9]. De notre étude il se dégage plusieurs facteurs de risque de surdosage: l’âge avancé, le sexe féminin, les trois premiers mois du traitement mais également une prise supérieure à 12 mois, les interactions médicamenteuses: AINS, amiodarone, antibiotiques, AAP, antifongiques, la présence d’une insuffisance cardiaque et d’une insuffisance rénale. Cependant d’autres facteurs, que nous n’avons pas trouvés, ont été rapportés dans la littérature à savoir le faible poids du patient et par conséquent l’indice de masse corporelle bas, les antécédents de surdosage, la présence d’une fièvre, une prise de plus de 7 médicaments. Des facteurs génétiques sont également impliqués, notamment la présence de mutation au niveau de gènes du cytochrome P450 2C9 et de la sous-unité 1 du complexe de la vitamine K époxyde réductase (VKORC1) [9, 17]. Des recommandations concernant les surdosages en AVK ont été remises à jour en 2008 (Tableau 3) [2]. Les moyens de corriger un surdosage en AVK sont: suspendre transitoirement l’AVK et attendre la réversibilité de l’effet anticoagulant de l’AVK qui est variable chez les patients. Le Sintrom a une réversibilité plus rapide (1 à 2 jours) que celle du Previscan (2 à 3 jours) du fait de sa demi-vie plus courte. La seule solution pratique étant alors de mesurer l’INR tous les jours après l’arrêt [2]; Administrer de la vitamine K qui permet une production endogène hépatique de facteurs coagulants. Plusieurs études ont montré que l’administration orale de 1 mg chez des patients ayant un INR entre 5 et 9, réduit significativement le temps de réversibilité de l’AVK et que l’administration d’une dose supérieure de vit K exposerait à une résistance pendant quelques jours, des doses supérieures (2 à 5 mg) sont cependant nécessaires en cas de surdosage important [18]. Les recommandations actuelles internationales proposent d’administrer la vitamine K à la dose de 5 à 10 mg [1]. La vitamine K peut être également administrée par voie intra-veineuse (IV). Mais par cette voie, elle doit être administrée lentement dans une perfusion en raison d’un risque de choc anaphylactique. Son effet est mesuré entre 6 à 8 heures après l’administration [1, 2]; Administrer du PPSB qui est un concentré de facteurs vitamino-K dépendants: Prothrombine (facteur II), Proconvertine (facteur VII), facteur Stuart (facteur X) et facteur antihémophilique B (facteur IX). En l’absence de PPSB qui n’est pas disponible en Afrique, le plasma frais congelé peut être utilisé. Le volume à perfuser est de 10 à 15 ml/Kg. Cependant le délai d’antagonisation est élevé et la qualité de la correction est de moins bonne qualité que celle obtenue avec un concentré de facteurs de coagulation, notamment sur le taux de facteur IX. De plus, la survenue d’effet secondaire lié à l’apport volumique important est fréquente [2, 5, 11].

**Tableau 3:** Mesures correctrices des surdosages en AVK en fonction de l’INR: recommandations HAS 2008

INR mesuré	INR cible entre 2 et 3	INR cible ≥ 3
4 ≤ INR < 6	-Saut d’une prise	- Pas de saut de prise
	-Pas d’apport de vit K	- Pas d’apport de vit K
6 ≤ INR < 10	-Arrêt du traitement	- Saut d’une prise
	-1 à 2 mg de vit K par VO	- Avis spécialisé recommandé pour discuter un traitement éventuel par 1 à 2 mg de vit K par VO
INR ≥ 10	-Arrêt du traitement -5 mg de vit K par VO	- Un avis spécialisé sans délai ou une hospitalisation est recommandé

## Conclusion

Les surdosages aux AVK sont une réalité en Afrique. Il existe des facteurs de risque qu’il convient de ne pas méconnaitre pour éviter ces situations et limiter le risque d’une hémorragie dont les conséquences peuvent être dramatiques voire fatales. L’éducation thérapeutique est primordiale. Il incombe alors aux professionnels de la santé de donner aux patients les moyens de gérer leur traitement en leur permettant d’acquérir les connaissances, les comportements et les gestes appropriés. L’utilisation de carnets dédiés au traitement anticoagulant expliquant de façon exhaustive le traitement et remis systématiquement à tout patient sous AVK serait d’un apport considérable à leur prise en charge.

### Etat des connaissances actuelle sur le sujet

- Peu d'études y ont été consacrées en Afrique subsaharienne et au Sénégal, aucune n'a été réalisée à ce sujet;
- Ceci fait qu'il manque des données épidémiologiques sur ces surdosages et sur leurs différents facteurs de risque.

### Contribution de notre étude à la connaissance

- Notre étude pourrait apporter l'expérience du Sénégal sur ces différents aspects, mais aussi, améliorer notre pratique quotidienne en agissant précocement sur ces différents facteurs, en insistant dans l'éducation thérapeutique de nos patients sur certains points susceptibles d'être à l'origine de surdosage, le tout pour assurer une meilleure prise en charge de nos patients.
